# Deficiency of protocadherin 9 leads to reduction in positive emotional behaviour

**DOI:** 10.1038/s41598-022-16106-5

**Published:** 2022-07-13

**Authors:** Masato Uemura, Tamio Furuse, Ikuko Yamada, Tomoko Kushida, Takaya Abe, Keiko Imai, Soichi Nagao, Moeko Kudoh, Katsuhiko Yoshizawa, Masaru Tamura, Hiroshi Kiyonari, Shigeharu Wakana, Shinji Hirano

**Affiliations:** 1 Laboratory of Cell Biology, Faculty of Medicine, Kansai Medical University, Shinmachi 2-5-1, Hirakata City, Osaka 573-1010 Japan; 2 Technology and Development Team for Mouse Phenotype Analysis, Japan Mouse Clinic, RIKEN BioResource Research Center, Tsukuba, Ibaraki 3050074 Japan; 3 Laboratory for Animal Resources and Genetic Engineering, RIKEN Center for Biosystems Dynamics Research, 2-2-3 Minatojima-minamimachi, Chuo-ku, Kobe, Hyogo 650-0047 Japan; 4 Laboratory for Motor Learning Control, RIKEN Brain Science Institute, Wako, Saitama 351-0198 Japan; 5 Laboratory of Environmental Science, Department of Innovative Food Sciences, School of Food Sciences and Nutrition, Mukogawa Women’s University, Nishinomiya, Hyogo 663-8558 Japan; 6 Present Address: Laboratory for Integrative Brain Function, Nozomi Hospital, Komuro 3170, Ina, Saitama 362-0806 Japan; 7 Present Address: Department of Neurophysiology, National Institute of Neuroscience, National Center of Neurology and Psychiatry, 4-1-1 Ogawa-Higashi, Kodaira, Tokyo 187-8502 Japan; 8 Present Address: Department of Animal Experimentation, Foundation for Biomedical Research and Innovation at Kobe, Kobe, 650-0047 Japan

**Keywords:** Cadherins, Cellular neuroscience, Development of the nervous system, Neural circuits, Amygdala

## Abstract

Protocadherin 9 (Pcdh9) is a member of the cadherin superfamily and is uniquely expressed in the vestibular and limbic systems; however, its physiological role remains unclear. Here, we studied the expression of Pcdh9 in the limbic system and phenotypes of *Pcdh9*-knock-out mice (*Pcdh9* KO mice). *Pcdh9* mRNA was expressed in the fear extinction neurons that express protein phosphatase 1 regulatory subunit 1 B (Ppp1r1b) in the posterior part of the basolateral amygdala (pBLA), as well as in the Cornu Ammonis (CA) and Dentate Gyrus (DG) neurons of the hippocampus. We show that the Pcdh9 protein was often localised at synapses. Phenotypic analysis of *Pcdh9* KO mice revealed no apparent morphological abnormalities in the pBLA but a decrease in the spine number of CA neurons. Further, the *Pcdh9* KO mice were related to features such as the abnormal optokinetic response, less approach to novel objects, and reduced fear extinction during recovery from the fear. These results suggest that Pcdh9 is involved in eliciting positive emotional behaviours, possibly via fear extinction neurons in the pBLA and/or synaptic activity in the hippocampal neurons, and normal optokinetic eye movement in brainstem optokinetic system-related neurons.

## Introduction

The protocadherin (Pcdh) family forms the largest subgroup in the cadherin superfamily; Pcdhs are divided into clustered- (α, β, γ) and non-clustered (δ)-types according to their genomic organisation^1–3^, and are known to be involved in the neural circuit formation, synaptogenesis, and neural activity. Pcdh9, a member of δ group, is uniquely expressed in various neural circuits, including the limbic, vestibular, and oculomotor systems^4^. The *Pcdh9* gene was reported to be a susceptibility gene for autism spectrum disorder and other psychiatric diseases^5^. Furthermore, it has been reported that Pcdh9 is involved in the cortical development and long-term social and object recognition^6^; however, the exact physiological role of Pcdh9 in the nervous system remains largely unknown.

Emotional behaviours are controlled by the limbic system that includes the amygdala and hippocampus. Amygdala, in particular, is critical for eliciting the emotional behaviours; the positive and negative emotional valences are combined in the basolateral amygdala (BLA), and the BLA sends this information to the output neurons in the central amygdala, leading to appetitive or non-appetitive behaviours^7^. It was reported that the neurons with expressed protein phosphatase 1 regulatory subunit 1 B (*Ppp1r1b*, also known as Dopamine- and cAMP-regulated phosphoprotein, Mr 32 kDa; DARPP-32)^+^neurons in the posterior part of the BLA (pBLA) and R-spondin 2 (Rspo2) in the anterior part of the BLA (aBLA) are involved in eliciting the appetitive and non-appetitive behaviours, respectively^7^.

Here, we examined the expression and distribution of Pcdh9 in the amygdala and hippocampus. We also determined Pcdh9 localisation in neurons and studied the phenotypes associated with neural circuit formation, neural functions, and behaviours using the *Pcdh9*-knock-out mice (*Pcdh9* KO mice). We revealed that *Pcdh9* was mainly expressed in Ppp1r1b^+^ neurons in the pBLA, and Cornu Ammonis (CA) and Dentate Gyrus (DG) neurons of the hippocampal formation; we found that the protein is often localised at the contact sites of neurites and synapses, along with other puncta over the cell surface. Histological analyses of the nervous system showed that the brains of the *Pcdh9* KO mice were largely normal, including projections of major neural pathways. However, neuronal defects such as larger ventricles and reduced synaptic density were detected in the hippocampus. Physiological analyses revealed that the *Pcdh9* KO mice had an abnormal optokinetic response (no changes were observed in the vestibular response). In the behavioural analyses, we found that the deficient mice tended not to approach novel objects, and the fear extinction was reduced during the recovery of fear. Our results suggest that Pcdh9 is involved in eliciting emotional behaviours, possibly via cell–cell interactions among the pBLA Ppp1r1b^+^ and hippocampal neurons, and in normal optokinetic eye movement in brainstem optokinetic system-related neurons.

## Results

### Expression of *Pcdh9* in the amygdala and hippocampus

**Figure 1:**
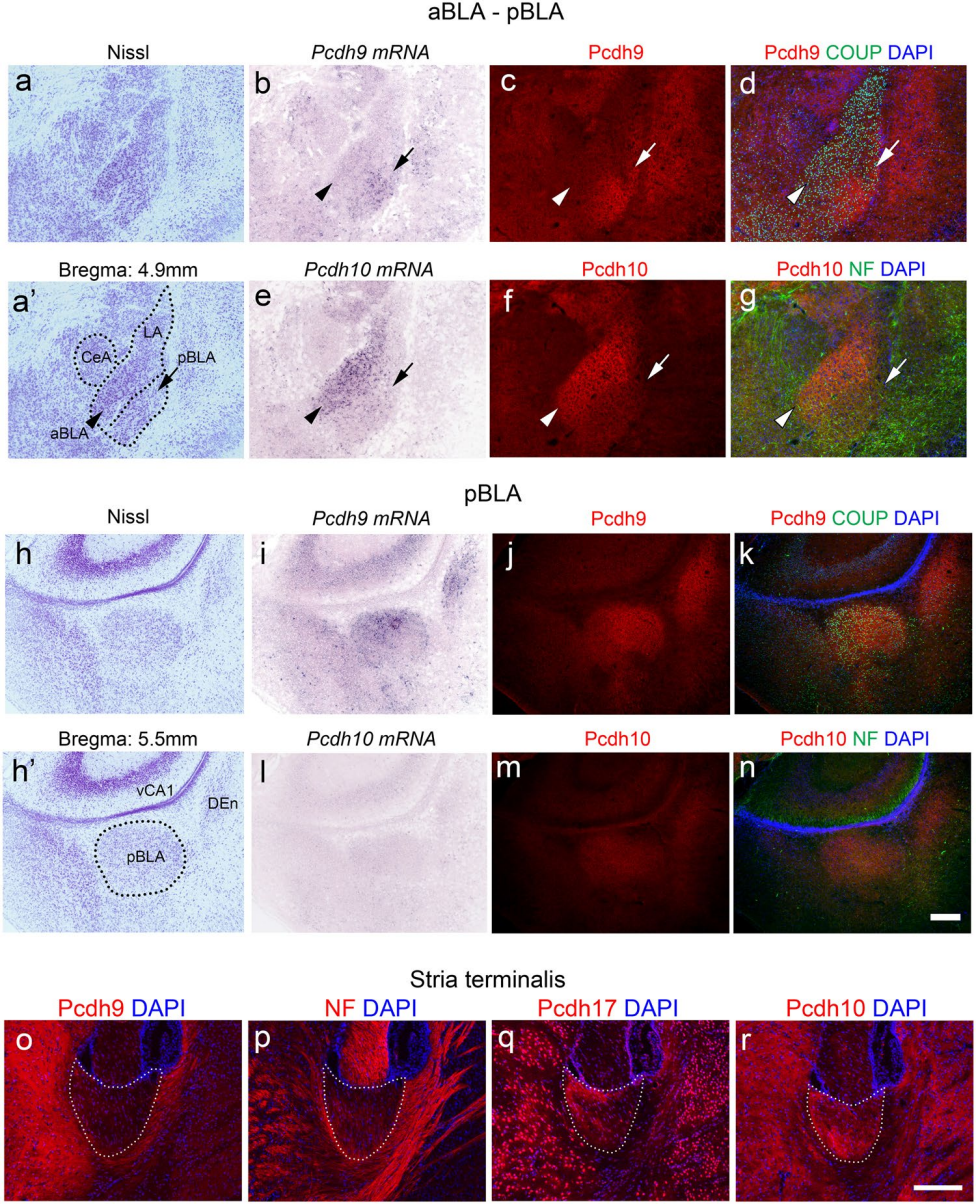
Expression and distribution of *Pcdh9* gene products in the amygdala and stria terminalis. (**a**–**n**) Expression and distribution of the *Pcdh9* gene products in the amygdala region in comparison to that of the *Pcdh10*. (**b**–**d**) and (**i–k**) Show the *Pcdh9* gene products in the aBLA and pBLA regions, respectively. Similarly, (**e**–**g**) and (**l**–**n**) Show the *Pcdh10* gene product in the aBLA and pBLA regions, respectively. (**o**–**r**) Staining of the stria terminalis with various anti-protocadherin antibodies. Most of the fibres in the stria terminalis were *Pcdh9* negative, whereas *Pcdh10* and *Pcdh17* were positive in the subsets of fibres. Abbreviation: NF, neurofilament. Scale bars, 200 μm in (**a**–**n**) and 100 μm in (**o**–**r**).

Previously, we reported that the Pcdh9 protein was distributed in various brain regions, including the vestibular and oculomotor systems^4^. Because *Pcdh9* KO mice showed abnormalities in emotional behaviours (see below), we focused on the expression of *Pcdh9* in the amygdala and related regions using in situ hybridization and immunohistochemistry (Fig. 1). Expression of *Pcdh9* mRNA was specifically detected in pBLA, but no or faint in aBLA (Fig. 1b,e,i,l). The distribution of the Pcdh9 protein was consistent with the mRNA expression patterns whereas no or faint signal could be detected in the anterior part of the BLA (aBLA) where the staining signal of Pcdh10 was strong (Fig. 1b,c,i,j). Although the Pcdh9 protein was broadly distributed, its co-localisation with synapsin I was limited (see Supplementary Fig. S1a–c online). Other amygdala regions, including the central amygdala, showed no Pcdh9-specific mRNA or protein (Fig. 1b). In the stria terminalis that contains fibres from the various amygdala regions, we could not observe prominent Pcdh9^+^ fibres (Fig. 1o).

In the brain regions that are known to be connected with the pBLA^8^, we observed a moderate signal in the ventral part of the CA1 (vCA1) of the hippocampus, while faint or no signals were observed in other regions such as the infralimbic cortical area (ILA) and medial part mediodorsal nucleus of the thalamus (MD) (Supplementary Fig. S2a; see Supplementary Fig. S1f, k online). In the hippocampal formation, *Pcdh9* was expressed in CA and DG neurons, but the staining of the Pcdh9 protein was weak (Supplementary Fig. S2b). Among the Pcdh9^+^ signals, a stronger signal was observed in the calbindin^+^ mossy fibres in the CA3 region (Supplementary Fig. S2b, e, h); most of the Pcdh9 staining did not overlap with that of synapsin I (Supplementary Fig. S2g–i) in this region.

### Identification of ***Pcdh9***^+^ neurons in the pBLA

**Figure 2:**
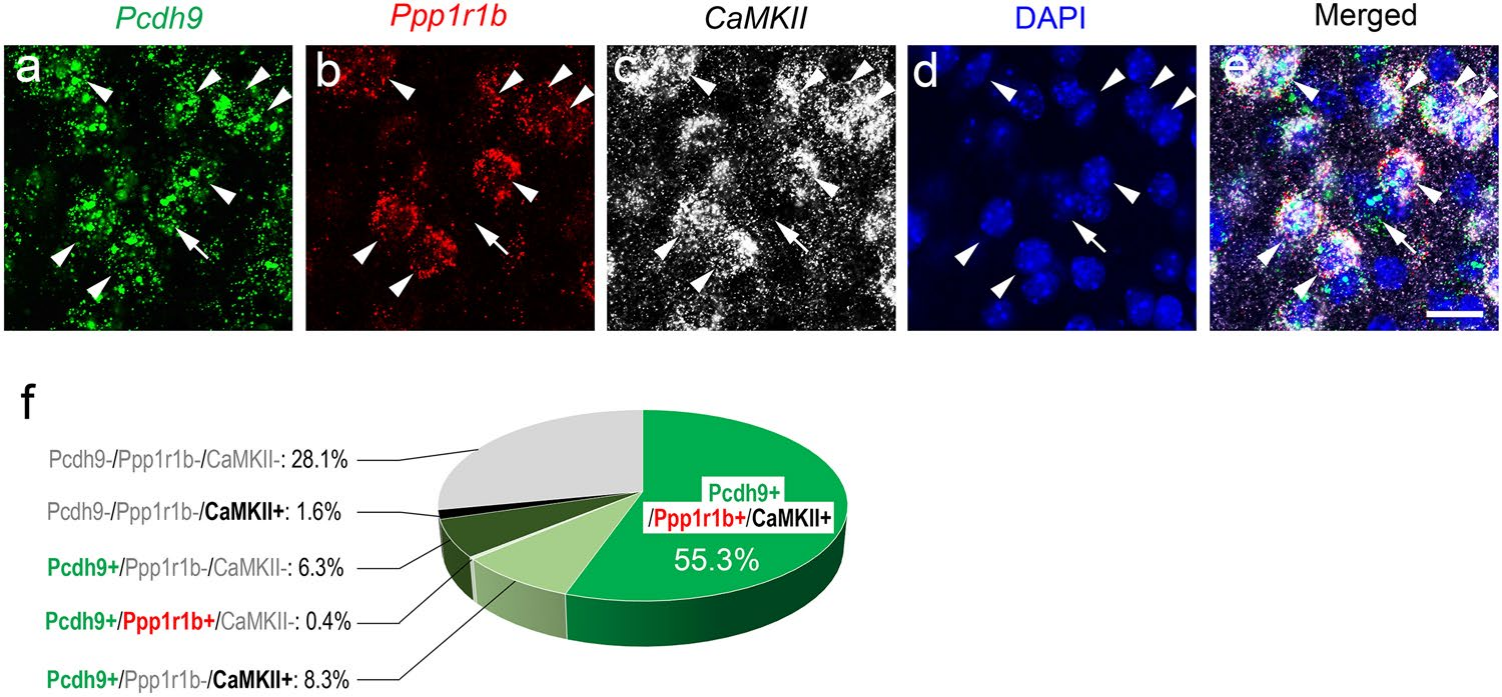
Identification of *Pcdh9*-expressing neurons. (**a**–**e**) Expression of *Pcdh9* mRNA; *Pcdh9* (**a**) was compared with *Ppp1r1b* (**b**) and *CamKII* (**c**) using multi-color in situ hybridization. Arrowheads indicate *Pcdh9*^+^ neurons that also expressed *Ppp1r1b*mRNA, whereas arrows indicate Pcdh9^+^ neurons that did not express *Ppp1r1b* mRNA. (**f**) Composition of pBLA neurons that expressed *Pcdh9*, *Ppp1r1b*, and/or *CamKII* in different combinations. Scale bar, 20 μm in (**a**–**e**).

To identify the cell type of *Pcdh9*^+^ neurons in the pBLA, we employed a multiple in situ hybridization method (Fig. 2a–e). Previously, it was reported that the “fear-extinction” neurons, which are involved in valence coding for appetitive behaviour, exist in the pBLA^7^. Since the fear recovery was reduced in the behavioural analysis of *Pcdh9* KO mice (see below), we compared the expression of *Pcdh9* with that of the *Ppp1r1b* (also known as *DARPP-32*; a marker of "fear-extinction" neurons) and calcium/calmodulin-dependent protein kinase II (*CamKII*; a marker of excitatory neurons). In the pBLA, *Pcdh9*^+^ neurons accounted for approximately 70% of the total neurons, and approximately 91% of the *Pcdh9*^+^ neurons were *CamKII*^+^ (*CamKII*^+^/total *Pcdh9*^+^), whereas approximately 8.5% were *CamKII*^-^ (*CamKII*^-^/total *Pcdh9*^+^) (Fig. 2f). *Ppp1r1b*^+^ neurons almost overlapped with that of the *Pcdh9*^+^ neurons (*Ppp1r1b*^+^/total *Pcdh9*^+^). These results suggest that a large proportion of *Pcdh9*^+^ neurons are excitatory fear extinction neurons, while only a small proportion are inhibitory neurons.

### Subcellular localization of the Pcdh9 protein in vitro

**Figure 3:**
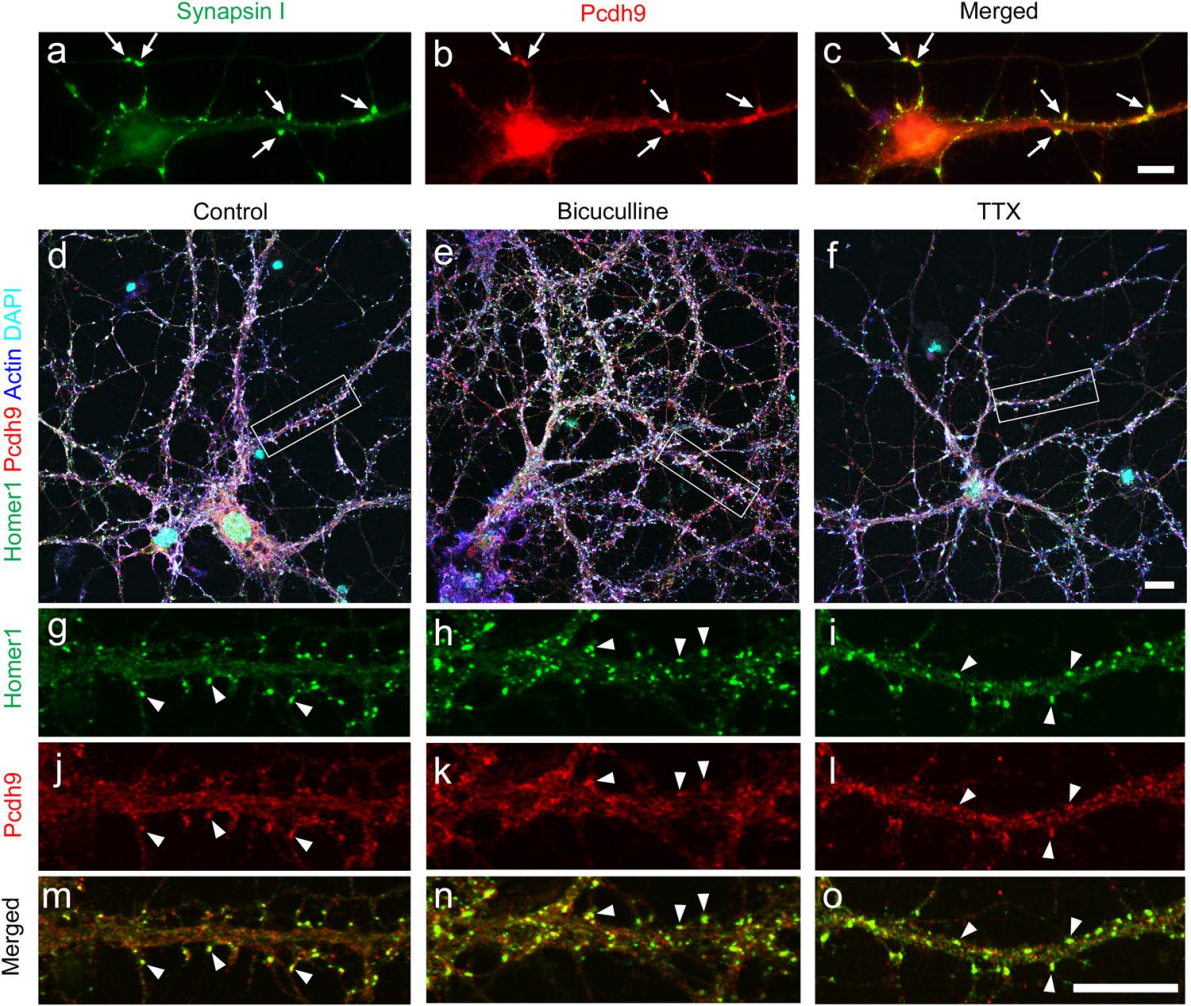
Localization of Pcdh9 proteins in cultured hippocampal neurons. (**a–c**) In 5-days in vitro (5-DIV) cultures, Pcdh9 protein was often localized at contact sites between neurites (arrows). (**d**–**o**) In 20-DIV cultures, Pcdh9 protein was often localized at synapses (arrowheads) in addition to other regions of neurites. Synaptic localization was not changed in normal medium (**d**, **g**, **j**, **m**), medium containing bicuculline (**e**, **h**, **k**, **n**), and TTX (**f**, **i**, **l**, **o**). Scale bars, 10 μm in (**a**–**c**), 10 μm in (**d–f**) and 10 μm in (**g–o**).

To understand the relationship of Pcdh9 with synapses, we examined the subcellular localisation of Pcdh9 protein in cultured hippocampal neurons. As reported previously with dorsal thalamus neurons^4^, we observed many puncta and/or clusters of Pcdh9 signals along neurites, growth cones, and contact sites in short cultures around 5 days in vitro (5DIV; Fig. 3a–c). In 14–21 DIV neurons, Pcdh9 protein was often co-localised with synapse markers at the contact sites among neurites (Fig. 3d,g,j,m). In addition to synaptic localisation, many Pcdh9^+^ puncta and clusters were distributed broadly along the axons. The subcellular localisation of Pcdh9 did not change in an activity-dependent manner following treatment of tetrodotoxin (TTX) and bicuculline (Fig. 3d–o).

### Defects of the nervous system in the *Pcdh9*-deficient mouse

To understand Pcdh9 roles in vivo, we generated *Pcdh9* KO mice and examined the phenotypes. Generally, the bodyweight of the KO mice was lower than that of the wild-type (wt) mice (see Supplementary Fig. S3c online). We could not find any major defects in various tissues, including the lungs, kidneys, and heart, using general histological examinations. The overall structure of the *Pcdh9* KO brain did not show gross defects; however, larger ventricles in the posterior region were observed than that of the wt mice (see Supplementary Fig. S4e, f, g online). The thickness of the cerebral cortex in the *Pcdh9* KO mice was identical to that of the wt mice (see Supplementary Fig. S5 online). Furthermore, we also could not find abnormalities in the major neural tracts (see Supplementary Fig. S6 online).

**Figure 4:**
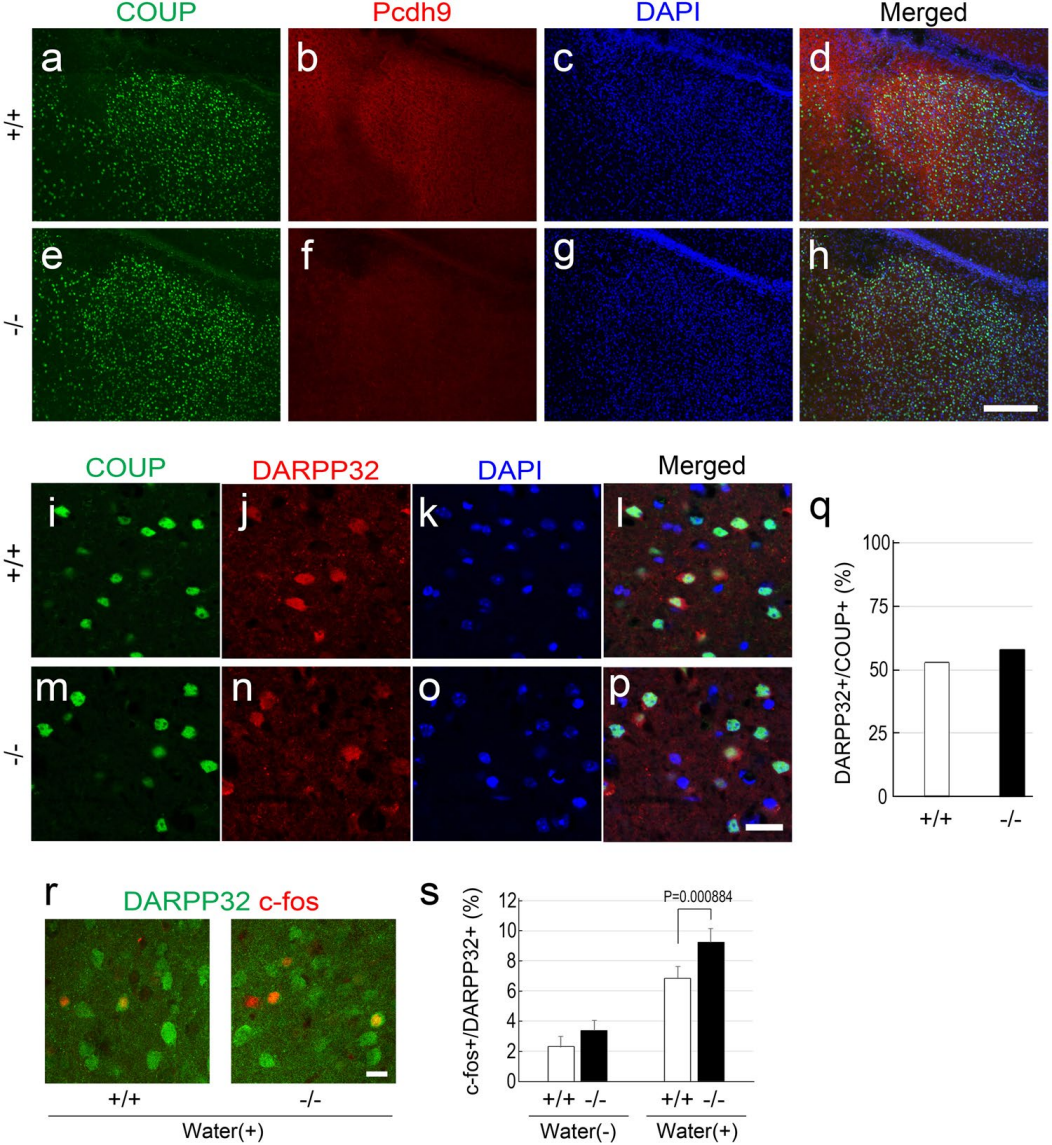
pBLA in the *Pcdh9* KO mice. (**a**–**h**) The size of pBLA, visualized with a marker of BLA neurons (COUP-TFII), was not affected in the KO mice. (**i**–**p**) Double staining of *COUP-TFII* and *DARPP-32* (*Ppp1r1b*), a marker of fear extinction neurons. (**q**) The number of *DARPP-32*^+^ neurons was not changed in the pBLA of the KO mice. (**r**) Double staining of *c-fos* (red) and *DARPP32* (green) after a water reward. (**s**) *c-fos* expression in the *DARPP-32*^+^ neurons was not different between the wt mice and the *Pcdh9* KO mice without a water reward, whereas it was significantly higher in the *Pcdh9* KO mice than that of the wt mice after a water reward. In the case without water reward, the numbers of c-fos^+^ and DARPP-32^+^ were counted with 10 sections from two wt mice and 12 sections from two Pcdh9 KO mice, whereas in the case with water reward, the numbers of c-fos^+^ and DARPP-32^+^ were counted with 28 sections from five wt mice and 30 sections from six Pcdh9 KO mice. Scale bars, 200 μm in (**a**–**h**) and 20 μm in (**i**–**p**, **r**).

**Figure 5:**
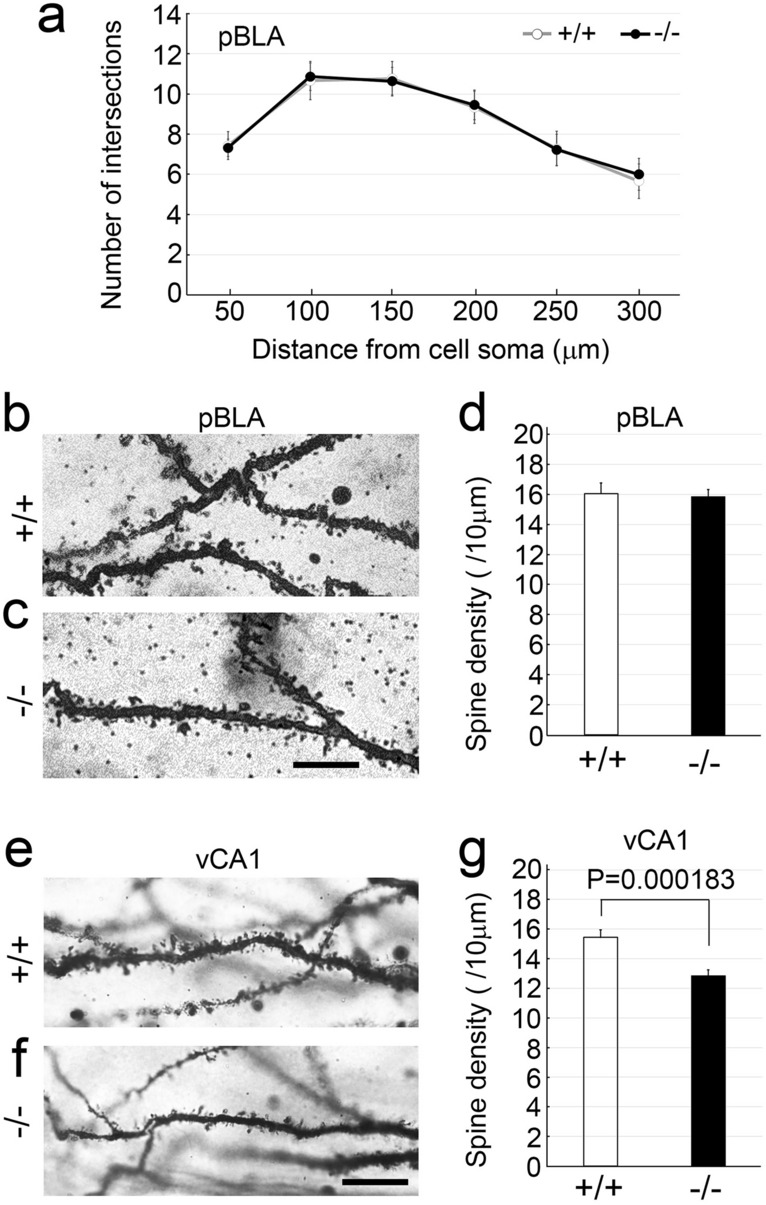
Morphological analyses of neurons in the *Pcdh9* KO mice with Golgi staining. (**a**) Statistics of dendritic arborisation of neurons in the pBLA. The number of branches from the primary dendrite was counted with 21 neurons from two wt mice and 22 neurons from four *Pcdh9* KO mice. Two-way ANOVA *p* = 0.76832. (**b**–**d**) The spine density of neurons in the pBLA was not changed in the *Pcdh9* KO mice. The number of spines was counted with 31 neurons from two wt mice and 65 dendrites from four *Pcdh9* KO mice. (**e**–**g**) Spine density in the vCA1 was significantly reduced in the *Pcdh9* KO mice. The number of spines was counted with 38 neurons from two wt mice and 94 neurons from four *Pcdh9* KO mice. Scale bars, 10 μm in (**b**, **c**) and 100 μm in (**e**, **f**).

In comparison to the wt mice, the size of pBLA was identical in *Pcdh9* KO mice (see Fig. 4a–h); further, no changes were observed in the number of *Ppp1r1b (DARPP-32)*^+^ neurons in the pBLA (Fig. 4i–q). Previously, it was reported that *Ppp1r1b*^+^ and "reward neurons" overlap significantly in the BLA, and are activated by rewards^9^. Hence, we examined *c-fos* expression in the *Ppp1r1b*^+^ neurons after a water reward. The results showed that a water reward induced higher *c-fos* expression in *Pcdh9* KO mice than that in the wt mice (Fig. 4r,s). Spine density and branching of the primary dendrites of the pyramidal neurons of the pBLA with conventional Golgi staining also did not show any detectable changes in the *Pcdh9* KO mice as compared to the wt mice (Fig. 5a–d).

In comparison to the wt mice, spine density was decreased in pyramidal neurons in the CA1 region, including the ventral part of the hippocampus in the *Pcdh9* KO mice (Fig. 5e–g). When the boutons of mossy fibres were visualised using the Thy-1 GFP transgene in the CA3 region, *Pcdh9* KO mice showed reduced bouton size in comparison to the wt mice (see Supplementary Fig. S7c, d, e online).

### Deficient of *Pcdh9* gene affected the optokinetic response in eye movement

**Figure 6:**
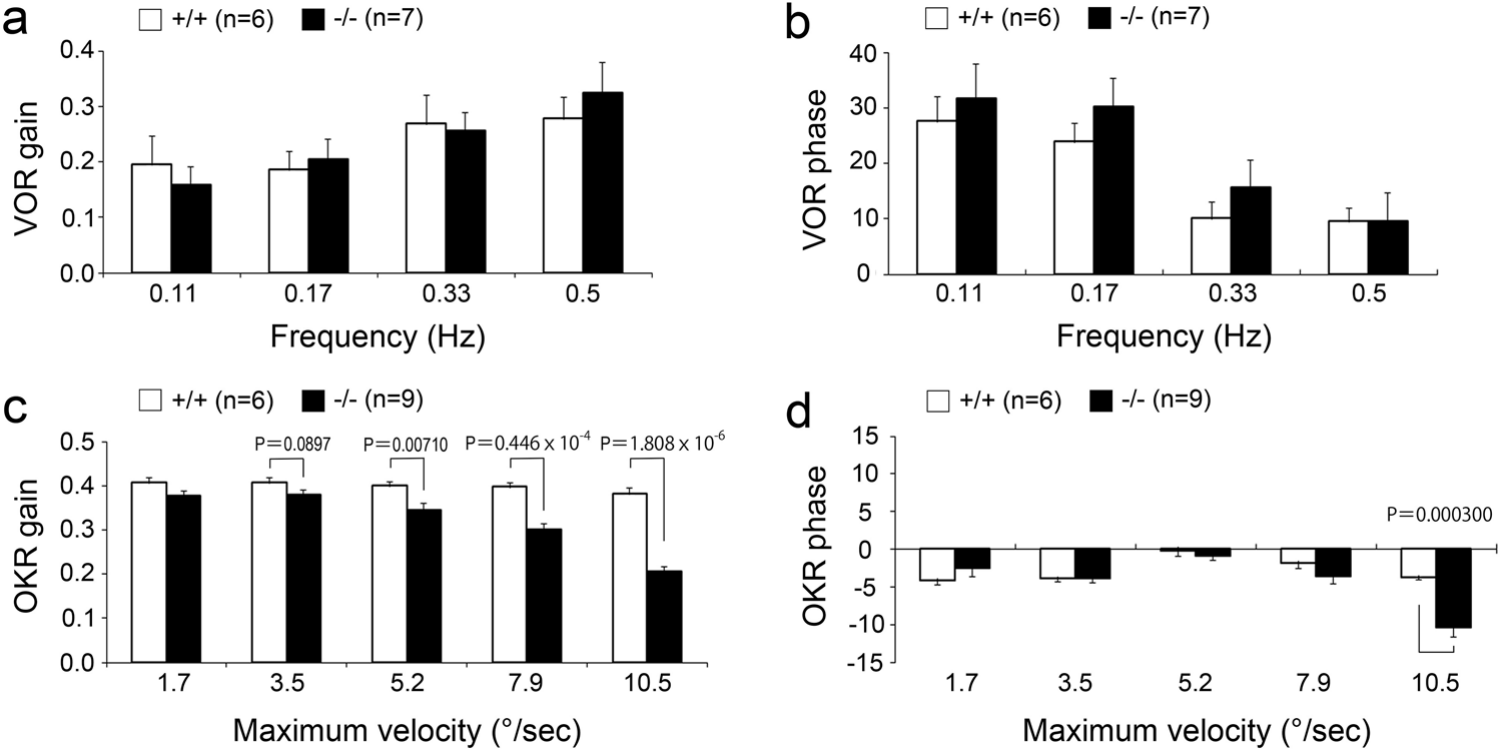
Abnormal optokinetic response (OKR) in the *Pcdh9* KO mice. (**a**, **b**) Vestibulo-ocular reflex (VOR) gain and VOR phase were not changed in the *Pcdh9* KO mice. (**c**, **d**) OKR gain and OKR phase were reduced at the higher maximum velocities in the *Pcdh9* KO mice. Scale bar, 2 mm.

Because *Pcdh9* is prominently expressed in the vestibular and oculomotor systems^4^, we first examined the sense of balance in *Pcdh9* KO mice. No changes were observed in gait, righting reflex, and contact righting reflex of the *Pcdh9* KO mice in comparison to the wt mice (Supplementary Table S1 online). Further, the auditory brainstem response was the same as the wt mice. We also examined the vestibular and optokinetic eye movement responses of *Pcdh9* KO mice according to a previously described method^10^. The results showed that the vestibulo-ocular reflex (VOR) was not affected in the gains and phases in *Pcdh9* KO mice (Fig. 6a,b). In contrast, the optokinetic response (OKR) was significantly affected in the gains and phases in the *Pcdh9* KO mice (Fig. 6c,d). A significant difference was seen (*P* < 0.05, Welch's T-test) in the OKR gains at relatively higher screen velocities (5.2–10.5 deg/s). Further, the OKR phase was delayed at 10.5 deg/screen oscillation in the *Pcdh9* KO mice.

### *Pcdh9* KO mice showed abnormal emotional behaviours

**Figure 7:**
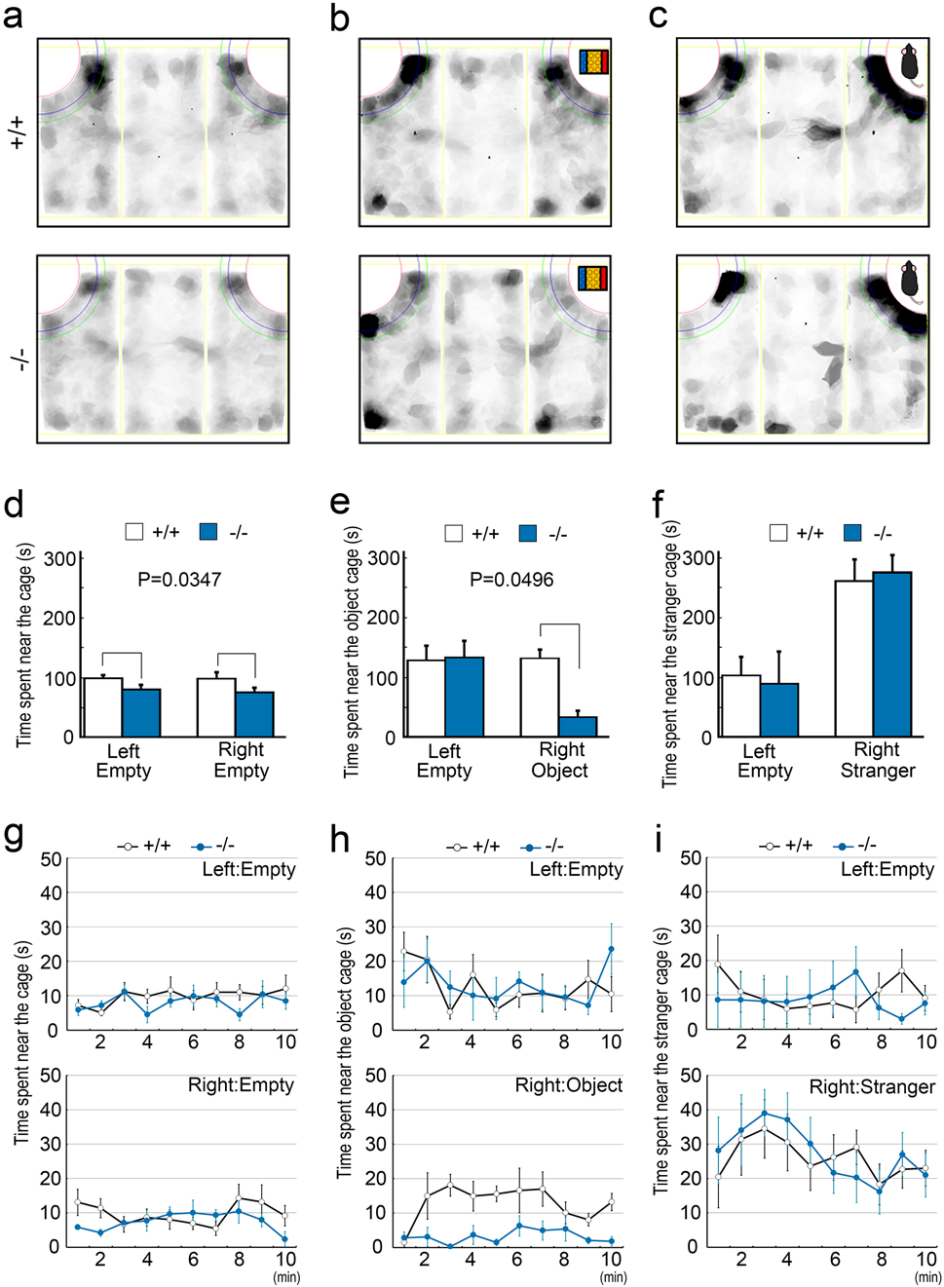
The reduced approach of the *Pcdh9* KO mice to a novel object. (**a**–**c**) Schematic diagrams of the three-chamber cages. The mouse behaviours were traced with shadows in each experiment. (**a**) Mice were habituated in the case with two empty chambers at the corners for 10 min before experiments. (**d**–**f**) Statistics (Two-way ANOVA) of the results in each experiment. (**d**) During habituation, the *Pcdh9* KO mice showed a tendency not to approach the empty chambers. (**e**) With a novel object in one side of the chambers, the approach of the *Pcdh9* KO mice to a novel object was significantly reduced. (**f**) In contrast, with a novel mouse in one side of the chambers, the *Pcdh9* KO mice behaved normally. (**g**–**i**) Time courses of staying time at each chamber. (**h**) *Pcdh9* KO mice tended to stay away from a novel object for all time. Scale bars, 100 μm in (**a**, **b**) and 10 μm in (**c**, **d**).

**Figure 8:**
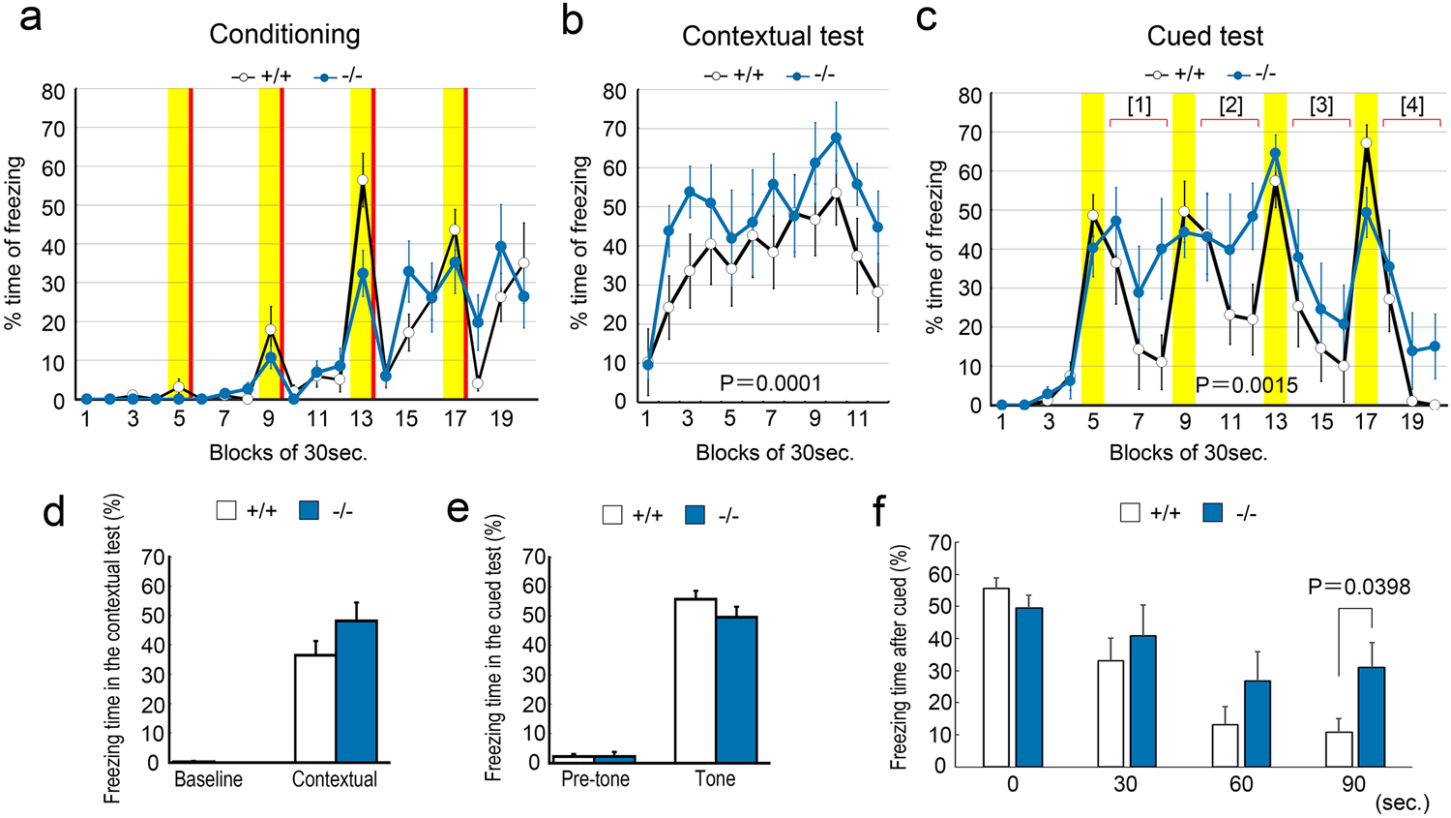
Reduced fear extinction of the *Pcdh9* KO mice. (**a**–**c**) Time course of freezing during conditioning, contextual, and cued tests in the conventional fear-conditioned memory test. The yellow shadows indicate the periods with tone stimulations whereas the red bars indicate the points of electric shocks. In both contextual and cued tests, a fear memory was established. In the recovery phases (Period [1]–[4]), freezing time of the Pcdh9 KO mice was significantly longer than that of the wt mice (2-way ANOVA). (**d**, **e**) In both contextual and cued tests, the total freezing time of the *Pcdh9* KO mice was not different from that of the wt mice. However, during the time course, the freezing time of the *Pcdh9* KO mice was longer than that of the wt mice in the contextual test with 2-way ANOVA analysis (**b**). (**f**) Comparison of the freezing time during the recovery phase of the cued test. The sum of the freezing times of each bin during four recovery phases (Period [1]–[4]) in the cued test is shown. The degree of freezing of the *Pcdh9* KO mice was significantly larger than that of the wt mice during the recovery from fear.

The behaviour of *Pcdh9* KO mice was evaluated according to the behavioural test battery of the Mouse Clinic (RIKEN BRC Web site)^11^, and the comparison was made with reference to the wt mice. In the light/dark transition test, we observed that the *Pcdh9* KO mice tended to delay entering the lightroom, but moved more frequently between bright and dark rooms (see Supplementary Fig. S8a, d online). In the open-field test, the *Pcdh9* KO mice showed normal behaviour in most parameters, except for the centre distance travelled (see Supplementary Fig. S8e, f online). We also performed Crawley’s social interaction test using three-chamber cages (Fig. 7). The *Pcdh9* KO mice approached the stranger mice in one of the chambers, similar to the wt mice (Fig. 7c,f,i). In contrast, we observed a significant reduction in the staying time of *Pcdh9* KO mice near the novel objects (Fig. 7b,e,h). In the home cage activity test, *Pcdh9* KO mice showed higher activity during the light period, although the total activity was identical to that of the wt mice (see Supplementary Fig. S9a–d online). In addition, *Pcdh9* KO mice showed lower activity transiently at the beginning of the dark period (see Supplementary Fig.S9f, g online). In the Y-maze test, we could not detect any differences in the alternation ratio and distance travelled between the wt and *Pcdh9* KO mice (see Supplementary Fig. S8g, h online). In the fear conditioning test (Fig. 8a–f), a fear memory was established in the *Pcdh9* KO mice, similar to the wt mice (Fig. 8d,e). However, fear was higher in the *Pcdh9* KO mice than that of the wt mice in the contextual cue (Fig. 8b), while the recovery from fear by cue was significantly delayed in the *Pcdh9* KO mice (Fig. 8c,f). In the pre-pulse inhibition test, *Pcdh9* KO mice generally showed normal responses, except for a stronger response at PP2 and a weaker response at PP2S (see Supplementary Fig. S10a online). Finally, we did not observe differences in the ratio of prepulse inhibition (see Supplementary Fig. S10b online).

Based on the observation that the *Pcdh9* KO mice stayed away from a novel object but not a novel mouse, we next performed a short-term object recognition test between the familiar and novel objects to examine the non-social specificity. We found a tendency of reduction in sniffing in *Pcdh9* KO mice (discrimination index ((time novel-time familiar)/(time novel + time familiar); *p* = 0.0908) and novel/familiar ratio (time novel/time familiar; *p* = 0.0391)) (see Supplementary Fig. S11 online). In addition, we performed a marble-burying test to examine whether fear was elevated in *Pcdh9* KO mice. During the test, the *Pcdh9* KO mice hid marbles in slightly smaller numbers (*p* = 0.119) (see Supplementary Fig. S12a online), but the timing of hiding the first marble was significantly delayed in the *Pcdh9* KO mice (see Supplementary Fig. S12b online).

## Discussion

We have previously reported that *Pcdh9* is expressed in various brain regions, including the vestibular and oculomotor systems^4^. In this study, we analysed the physiological role of Pcdh9 using the *Pcdh9* KO mice. We did not observe any major morphological abnormalities in the nervous system of the *Pcdh9* KO mice except for the wider ventricles. Previously, Bruining et al. produced a similar null *Pcdh9* KO mouse line and reported a reduction in the thickness of the cortex, including the somatosensory cortex^6^. However, we did not observe a reduction in the thickness of the cortex in our *Pcdh9* KO mice. One possible reason could be related to the difference between the genomes; our *Pcdh9* KO mice lacked the neomycin resistance gene that might have affected neural cells in *Pcdh9* KO mice developed by Bruining et al.

We did not observe clear abnormalities in the balance of walking in *Pcdh9* KO mice. Since most neurons express several cadherins, it is possible that they compensate for the lack of Pcdh9 in the vestibular system. In contrast, we observed abnormal OKR (but not VOR) in *Pcdh9* KO mice, suggesting that the OKR-specific neural circuits, including the brainstem accessory optic tract (AOT) and nucleus reticularis tegmenti pontis (NRTP), which specifically relay OKR to the extraocular muscle motor neurons via the vestibular nuclei in rodents^12–14^, might have some defects in *Pcdh9* KO mice. Hence, Pcdh9 may be involved in neural connections and/or synapse formation in these circuits.

Few abnormalities were detected in the *Pcdh9* KO mice during the behavioural analyses; although general fear did not increase in the light–dark transition, open field, and Y-maze tests, the fear extinction was significantly delayed in the *Pcdh9* KO mice during the recovery from the fear induced in the fear-conditioned memory test. It is reported that *Ppp1r1b*^+^ "fear-extinction" neurons exist in the pBLA, and the decision of appetitive or nonappetitive behaviour depends on the balance of positive and negative emotions via valence coding in the amygdala where the *Ppp1r1b*^+^ neurons in the pBLA led to a positive emotional value, while the *Rspo2*^+^ neurons in the aBLA led to a negative emotional value^7,15^. Expression of *Pcdh9* in *Ppp1r1b*^+^ neurons in the pBLA indicates that the deficiency of *Pcdh9* may cause abnormalities in fear-extinction neurons via a lack of Pcdh9-mediated cell–cell interactions. However, we also observed that the *c-fos* expression was significantly higher in *Ppp1r1b*^+^ neurons of *Pcdh9* KO mice than that of the wt mice after a reward. Since it was reported that *Ppp1r1b*^+^ neurons and reward neurons functionally overlap significantly in the pBLA^9^, the result did not seem consistent with the hypothesis that the activation of *Ppp1r1b*^+^ neurons is involved in fear extinction. We were not able to explain this contradiction; however, it is possible that they are not identical although the fear extinction process and the reward system significantly overlap.

Another interesting phenotype of *Pcdh9* KO mice is a significantly reduced approach to a novel object, but not to a novel mouse (Fig. 7). This observation is consistent with the results of the object recognition test and the delay in the starting time to hide marbles in the marble-burying test (Supplementary Figs. S11, S12 online). It is difficult to discriminate whether the reduction in approach is a result of avoidance with fear or ignorance without interest, but it is possible that fear was elevated in the *Pcdh9* KO mice via a defect in fear extinction. Because the fear of novel mice seemed not elevated, fear extinction may be specific to novel objects via proper recognition of objects.

Bruining et al. reported various phenotypes of their Pcdh9 KO mice, including abnormal cortical development, abnormal long-term social and object recognition, hyperactivity, and abnormal sensorimotor functions^6^. We found differences in phenotypes such as cortical development and emotional behaviour between Bruining et al. and our study. These differences may be due to the different conditions and sensitivity of analyses in these studies. Further research is needed to reveal the exact function of Pcdh9 in the nervous system. Since Pcdh9 is expressed in various brain regions, it is likely involved in various neural functions including fear extinction, OKR, cognitive function, and sensory processing.

In this study, we could not elucidate the mechanism of Pcdh9 involvement in the fear extinction neural circuits. Although the synaptic localization of Pcdh9 is limited in tissue section, Pcdh9 is likely to be involved in the neural connections because of its clear localization at cell–cell contact sites and synapses in neural culture. Further, if the Pcdh9 protein function as homophilic adhesion molecules, the targets of *Ppp1r1b*^+^ neurons should express Pcdh9. Among the fear extinction circuits, *Pcdh9* is expressed in vCA1, whereas IL, aBLA, and CeL are faint or negative. Thus, Pcdh9 may be involved in the long projection between the pBLA and vCA1. In addition, because of the localisation of Pcdh9 protein at the synapse in wt neuron cultures, and the reduction in synapse number in the KO CA1, Pcdh9 is likely to be involved in the earlier phase of synapse formation, and it might be decreased during maturation of the synapse. On the other hand, Pcdh9 puncta were distributed over the cell surface outside the synaptic region in cultured neurons and were also abundant outside the synaptic region in mature neural tissue. Therefore, Pcdh9 might have some unknown functions in addition to synapse formation. To explore some of the underlying mechanisms of Pcdh9-mediated neural functions and associated phenotypes identified in this study, further studies are needed.

## Material and methods

### Animals

ICR mice (Japan SLC) were used for in situ hybridization, immunohistochemical analyses, and primary cell cultures. To analyse the phenotypes of the *Pcdh9* KO mice, we used the wt siblings of the c57BL/6N background as controls.

To generate *Pcdh9* null mice (accession no. CDB0920K: http://www2.clst.riken.jp/arg/mutant%20mice%20list.html), the 2nd exon of the Pcdh9 gene, that encodes the large part of the molecule (1–1016 amino acids), was replaced with a neomycin resistant gene flanked with loxP sequence in the TT2 ES cell lines with conventional homologous recombination^16^ (see Supplementary Fig. S2a online). Homologous recombinant ES cells were injected into the ICR 8-cell stage embryos to generate chimeric mice. The resulting heterozygote mice derived from the targeted ES cells were backcrossed with a c57BL/6N strain for more than 10 generations. The neomycin gene was then removed from the genome by crossing it with Cre-expressing mice (B6.Cg-Tg(CAG-Cre) CZ-MO2Osb mouse line, RIKEN RBRC01828). We confirmed the targeted deletion of the 2nd exon of the *Pcdh9* gene by sequencing the targeted region and the boundaries between the vector arms and the genome. We also confirmed the loss of Pcdh9 protein in the KO mice by Western blot analysis (see Supplementary Fig. S1b online). After back-crossing with C57BL/6N for several generations, we used this heterozygote line to obtain homozygotes. For genotyping with PCR, we used two pairs of primers; TCTGGGCAAATGCAACAAGC and GAAGCCCCAGGAGCAGAATTG to detect the KO allele, and CGAGCTGGAGGAACAGGGTATAGG and GAAGCCCCAGGAGCAGAATTG to detect wt allele. These mice together with the wt mice were used for all the phenotypic analyses, except for the physiological tests for vestibular and optokinetic responses, where we used *Pcdh9* KO mice with a neomycin-resistant gene. To visualise mossy fibre boutons in the CA3 region, Thy1-GFP (line M) transgenic mice (Jackson Laboratory No: 007788, Tg (Thy1-EGFP) MJrs/J) were crossed with *Pcdh9* KO mice to obtain *Thy1-GFP/PCDH9* double mutant mice.

All animals were kept under specific-pathogen-free (SPF) conditions until experimental use. All procedures described here were reviewed and approved by the Institutional Animal Care and Use Committee of Kansai Medical University and the RIKEN Tsukuba, Wako, and Kobe branches, and experiments were performed under the institutional guiding principles for the care and use of laboratory animals. All methods are reported in accordance with ARRIVE guidelines (https://arriveguidelines.org).

### Preparation of brain sections and slices

Mice of various ages were deeply anaesthetised and transcardially fixed with 4% paraformaldehyde solution in HEPES-buffered balanced salt solution (HBSS). The brains were post-fixed in 4% paraformaldehyde (PFA) solution at 4 °C for 4–24 h depending on the mouse age. After fixation, the solution was replaced with a 20% sucrose solution for cryoprotection. The brains were frozen in embedding medium (Sakura, 4583). Serial brain sections were made at 14 μm thickness using a cryostat (Leica CM3050S) at − 20 °C, and they were attached onto the Poly-l-Lysine (PLL)-coated glass slides. To prepare brain slices, the fixed brains were sliced to 150 μm thickness using a vibratome (Dosaka EM, Japan, DTK-3000 W).

### Immunohistochemistry

Brain sections and slices were permeabilised using 0.25% Triton-X-100 in 50 mM Tris-buffered salt solution containing 1 mM Ca^2+^ (TBS-Ca) and washed with the TBS-Ca. After blocking with 5% skim milk in TBS-Ca, the following primary antibodies were applied in the blocking solution overnight at 4 °C. To detect Pcdh9, we used a 2D3 rat monoclonal antibody (mAb)^4^. Rat anti-OL-protocadherin (also known as Pcdh10; mAb 5G10) mAb and anti-Pcdh17 polyclonal antibodies (pAbs) have been previously described^17,18^. Mouse mAb 2H3 against neurofilament was obtained from the Developmental Studies Hybridoma Bank (University of Iowa). Other primary antibodies (and dilutions) used were: mouse anti-FoxP2 (Millipore, MAB415, 1:500), mouse anti-COUP-TFII (Perseus Proteomics, PP-H7147-00, 1:400), mouse anti-calbindin (Swant, CB300, 1:200), rabbit anti-DARPP-32 (Santa Cruz Biotechnology, sc11365, 1:500), rabbit anti-c-fos (Abcam, ab208942, 1:1000), rabbit anti-Synapsin I (Millipore, AP1543P, 1:1,000), rabbit anti-Homer (Synaptic Systems, 160003, 1:400). Primary antibodies were detected with appropriate Alexa488- or Alexa555-conjugated (Thermo Fisher A11029, A11034, A21424, and A21429) or Cy3-conjugated antibodies (Chemicon, AP183C, or AP189C, 1:500) as secondary antibodies. Actin filaments in the cultured neurons were visualized using Alexa647 conjugated phalloidin (Thermo Fisher, A22287).

### In situ hybridization

Conventional in situ hybridization was performed according to Suzuki et al.^19^ We synthesised digoxigenin-labelled sense- and antisense RNA probes for *Pcdh9* and *Pcdh10* using a DIG RNA labelling kit (Merck 11175025910). The probes corresponded to the 800 bp cDNA fragment (2680–3480 bp, MGC189939) of *Pcdh9* and 900 bp cDNA fragment of *Pcdh10*^20^. Multicolour fluorescent in situ hybridization (FISH) was performed using the RNAscope Fluorescent Multiplex R Kit (Advanced Cell Diagnostics, ACDBio). *Pcdh9* (Cat#524921), *Ppp1r1b* (Cat #405901), *Esr1* (Cat #432861), and *CaMkIIa* (Cat#445231) FISH probes were obtained from ACDBio. Single-plane and z-series FISH images were acquired using a confocal microscope (LSM700, Carl Zeiss).

### Micro Computed Tomography (CT) imaging

Micro-CT imaging was performed using Inveon (Siemens, Germany). CT scanningwas performed according to Zikmund et al.^21^. After transcardial perfusion with 4% PFA, the mouse brains were dissected from the skull and post-fixed with 4% PFA overnight at 4 °C. The brains were impregnated with a 1% iodine and 95% ethanol solution for 24 h after dehydration using a methanol series. Through the hydration step with a methanol series, excess iodine was washed, and brain samples were embedded in a 1% agarose block. Micro-CT scans were performed at 50 kV acceleration voltage and 500 μA tube current conditions. CT images were acquired at15.83 μm pixel size under 2000 ms exposure time without binning. Tomographic reconstruction was performed using OsiriX software (Newton Graphics, Inc., Japan). Masking of target neural tissue for 3D reconstruction and volume analysis of brain structures was carried out using Mimics software (Materialise, Belgium).

### Primary hippocampal neuron culture

Primary cultures of hippocampal neurons were performed according to a conventional method^22^. Briefly, hippocampal tissue dissected from the E17.5 mouse cortex was treated with 0.25% trypsin solution and then triturated with calcium- and magnesium-free HEPES buffered salt solution (HCMF). Hippocampal neurons were plated on PLL or laminin-coated glass coverslips at a density of 3.0–6.0 × 10^5^ cells/mL in neurobasal medium (GIBCO) supplemented with B27 (GIBCO), 2 mM L-glutamine, and 20 µg/mL gentamicin (GIBCO). For immunochemical analysis, neurons were fixed with 2% PFA and 4% sucrose in HBSS solution for 5 min at 37 °C. Neurons were permeabilised with 0.25% Triton-X-100 in TBS-Ca solution for 10 min and then blocked with 5% skim milk for 1 h. The neurons were incubated with primary and secondary antibodies. Fluorescent images were taken using a fluorescence microscope (Zeiss Axioplan and Axioscope) and a confocal microscope (Olympus FV3000).

### Analysis of spine density and dendritic arborization in the Golgi-stained brain

We performed the Golgi staining of adult brains using a Rapid Golgi Stain kit (FD Neuro Technologies), according to the manufacturer's protocol. The brains were impregnated with kit solutions and then sliced at 200 μm thickness using a cryostat at − 25 °C. The slices were mounted on gratin-coated slide grasses (FD Neuro Technologies). Tissue staining was performed using the kit solutions and mounted on glass slides after washing. Z-stack images of dendritic spines of neurons stained with Golgi solutions were obtained using a BZ-9000 microscope (Keyence, Japan) with a 100 × objective lens. Spine density and dendritic arborisation of neurons were analysed using ImageJ software.

### Physiological and behavioural tests

c-*fos* expression after a reward was examined according to the method described previously^9^. Acute water deprivation was performed against wt and mutant mice overnight, and then the mice were supplied with a sufficient amount of water using a conventional bottle the next early morning. The mice were perfused with 4% PFA solution at 90 min after the first access to water. *c-fos* and *DARPP-32* expressing neurons were visualised by immunostaining in the pBLA region, and the percentage of positive *c-fos* was calculated in the *DARPP-32* positive neurons.

Optokinetic response (OKR) and vestibulo-ocular reflex (VOR) were analysed as described before^10^.

All behavioural tests with male mice were performed at the Japan Mouse Clinic of RIKEN BRC. A battery of the following seven tests was carried out for comprehensive behavioural phenotyping: light/dark transition test (6-weeks old), open-field test (7-weeks old), Crawley’s social interaction test (3-chamber test, 9-weeks old), home-cage activity test (10 to 11-weeks old), Y-maze test (12-weeks old), fear conditioning test (13-weeks old), and pre-pulse inhibition test (14-weeks old). The results of the behavioural tests are sensitive to prior experience^23^. Therefore, to suppress the effects of prior experience, these tests were started from the least invasive test in the battery. For all the tests in the battery, mice were transferred from their husbandry room to the testing room for at least 30 min before the experiment. Seven males of each genotype were used as subjects in the test battery. We performed these behavioural analyses as described previously, with slight modifications^24^, and the detailed procedures are disclosed on the website^11^. In addition, we performed additional tests using another batch of male mice (n = 16), including the open field, object recognition, and marble-burying tests. The marble-burying and the object recognition tests were performed as previously reported with modifications^24,25^.

## Supplementary Information

Supplementary Information.

## Data Availability

The datasets generated during and/or analysed during the current study are available from the corresponding author on reasonable request.

## References

[1] Hirano S, Takeichi M (2012). Cadherins in brain morphogenesis and wiring. Physiol. Rev..

[2] Weiner KMMJA, Suzuki ST, Hirano S (2016). The cadherin superfamily.

[3] Jontes JD, Suzuki ST, Hirano S (2016). The cadherin superfamily.

[4] Asahina H, Masuba A, Hirano S, Yuri K (2012). Distribution of protocadherin 9 protein in the developing mouse nervous system. Neuroscience.

[5] Hirano S, Imai-Okano K, Suzuki ST, Hirano S (2016). The cadherin superfamily.

[6] Bruining H (2015). Genetic mapping in mice reveals the involvement of Pcdh9 in long-term social and object recognition and sensorimotor development. Biol. Psychiatry.

[7] Kim J, Pignatelli M, Xu S, Itohara S, Tonegawa S (2016). Antagonistic negative and positive neurons of the basolateral amygdala. Nat. Neurosci..

[8] Hintiryan H (2021). Connectivity characterization of the mouse basolateral amygdalar complex. Nat. Commun..

[9] Zhang X, Kim J, Tonegawa S (2020). Amygdala reward neurons form and store fear extinction memory. Neuron.

[10] Shutoh F, Ohki M, Kitazawa H, Itohara S, Nagao S (2006). Memory trace of motor learning shifts transsynaptically from cerebellar cortex to nuclei for consolidation. Neuroscience.

[11] https://ja.brc.riken.jp/lab/jmc/mouse_clinic/en/pipeline_en/pipeline_02.htm#p2-00

[12] Miyashita Y, Ito M, Jastreboff PJ, Maekawa K, Nagao S (1980). Effect upon eye movements of rabbits induced by severance of mossy fiber visual pathway to the cerebellar flocculus. Brain Res..

[13] Kano M, Iino K, Maekawa K, Kano MS (1991). Optokinetic response of cells in the nucleus reticularis tegmenti pontis of the pigmented rabbit. Exp. Brain Res..

[14] Nagao S (2021). Ocular reflex adaptation as an experimental model of cerebellar learning: In memory of Masao Ito. Neuroscience.

[15] Kim J, Zhang X, Muralidhar S, LeBlanc SA, Tonegawa S (2017). Basolateral to central amygdala neural circuits for appetitive behaviors. Neuron.

[16] Yagi T (1993). A novel ES cell line, TT2, with high germline-differentiating potency. Anal. Biochem..

[17] Aoki E, Kimura R, Suzuki ST, Hirano S (2003). Distribution of OL-protocadherin protein in correlation with specific neural compartments and local circuits in the postnatal mouse brain. Neuroscience.

[18] Hayashi S (2014). Protocadherin-17 mediates collective axon extension by recruiting actin regulator complexes to interaxonal contacts. Dev. Cell.

[19] Suzuki SC, Inoue T, Kimura Y, Tanaka T, Takeichi M (1997). Neuronal circuits are subdivided by differential expression of type-II classic cadherins in postnatal mouse brains. Mol. Cell Neurosci..

[20] Hirano S, Yan Q, Suzuki ST (1999). Expression of a novel protocadherin, OL-protocadherin, in a subset of functional systems of the developing mouse brain. J. Neurosci..

[21] Zikmund T, Novotná M, Kavková M, Tesařová M, Kaucká M, Szarowská B, Adameyko I, Hrubá E, Buchtová M, Dražanová E, Starčuke Z, Kaiser J (2017). High-contrast differentiation resolution 3D imaging of rodent brain by X-ray computed microtomography. JINST.

[22] Togashi H (2002). Cadherin regulates dendritic spine morphogenesis. Neuron.

[23] Crawley JN (2008). Behavioral phenotyping strategies for mutant mice. Neuron.

[24] Kim K (2019). Autophosphorylation of F-actin binding domain of CaMKIIbeta is required for fear learning. Neurobiol. Learn. Mem..

[25] Deacon RM (2006). Digging and marble burying in mice: Simple methods for in vivo identification of biological impacts. Nat. Protoc..

[26] Hirano, S. *et al.* Deficient of autism susceptibility gene Protocadherin 9 leads to abnormalities in vestibular response and emotional behavior in mice. *Annual meeting of the Society for Neuroscience* (*Neuroscience 2017)*, 651.614 (2017).

