# Supplementary material for: Deficiency of protocadherin 9 leads to reduction in positive emotional behaviour
Source: Sci Rep. 2022 Jul 13;12:11933. doi: 10.1038/s41598-022-16106-5 (PMC9279467; doi:10.1038/s41598-022-16106-5)
Supplement: Supplementary file 1 — Supplementary Information. [file 41598_2022_16106_MOESM1_ESM.pdf]

Uemura et al.   Supplementary Data

Supplementary Table S1.

Summary of physiological and behavior phenotypes of *Pcdh9* KO mice. n.s., no statistical difference ($p>0.0$); S, statistical difference ($P<0.05$).

test	gender	phenotype	Figures
Gait	male, female	ns$	
Righting reflex	male, female	ns	
Contact righting reflex	male, female	ns	
Vestibulo-ocular reflex (VOR)	male, female	ns	Fig.6
Optokinetic response (OKR)	male, female	S	Fig.6
Light/dark transition test	male	ns	Supplementary Fig.S8
Open-field test	male	S	Supplementary Fig.S8
Crawley social interaction test	male	S	Fig.7.
Home-cage activity test	male	S	Supplementary Fig.S1
Y-maze	male	ns	Supplementary Fig.S8.
Fear conditioning test	male	ns	Fig.8
Pre-pulse inhibition test	male	S	Supplementary Fig.S10
Object recognition test	male	S	Supplementary Fig.S11
Marble burying test	male	ns	Supplementary Fig.S12

$One out of 10 homozygous mice was abnormal.

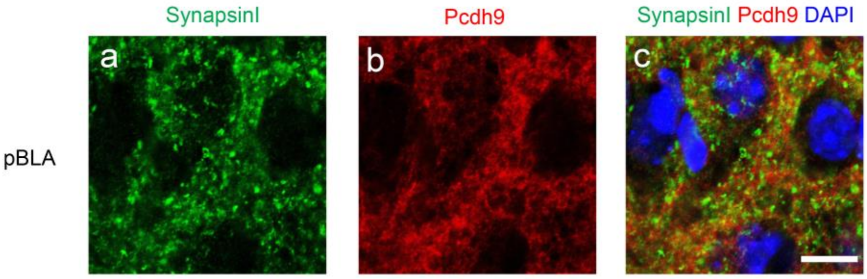

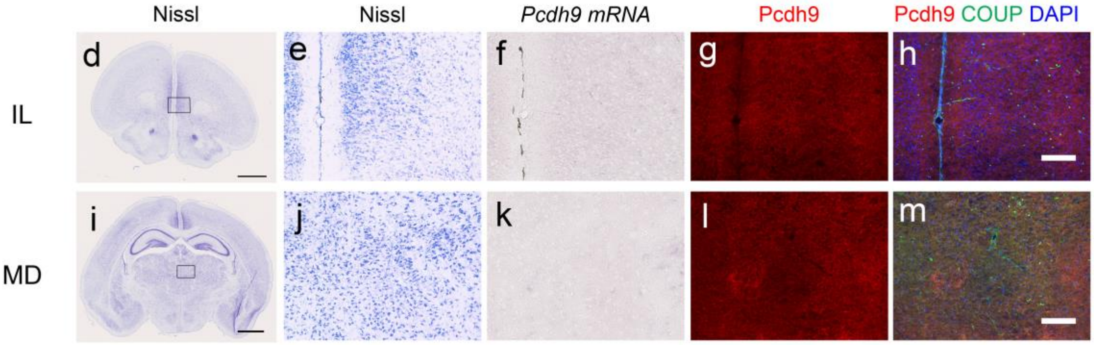

Supplementary Fig. S1 Expression and localization of *Pcdh9* gene product in the pBLA

(a-c) Localization of Pcdh9 protein in the pBLA with a comparison of synapsin I. (d-m) Expression of *Pcdh9* mRNA and distribution of Pcdh9 protein in the infralimbic cortex (IL) and mediodorsal thalamic nuclei (MD). Scale bars, 10µm in (a-c), 1mm (d, i), 100µm in (d-h) and 100µm in (i-m).

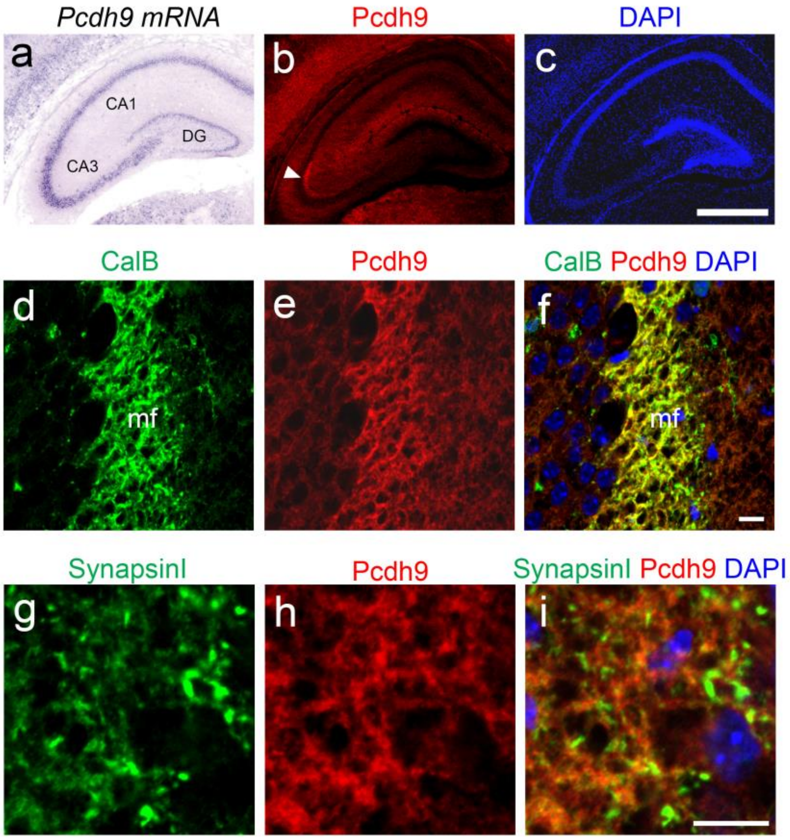

Supplementary Fig. S2 Expression and distribution of *Pcdh9* gene products in the hippocampus.

(a-c) Lower magnification of the hippocampal region where *Pcdh9* mRNA was expressed in the CA1 and DG prominently but the staining of the Pcdh9 protein was rather weak and diffuse. (d-f) Enlargement of the stained images with anti-Pcdh9 mAb in calbindin^{+} mossy fibres of the CA3 region. (g-i) Localization of Pcdh9 protein in the CA3 region in comparison to that of synapsin I. Scale bars, 500µm in (a-c), 10µm in (d-f) and 10µm in (g-i).

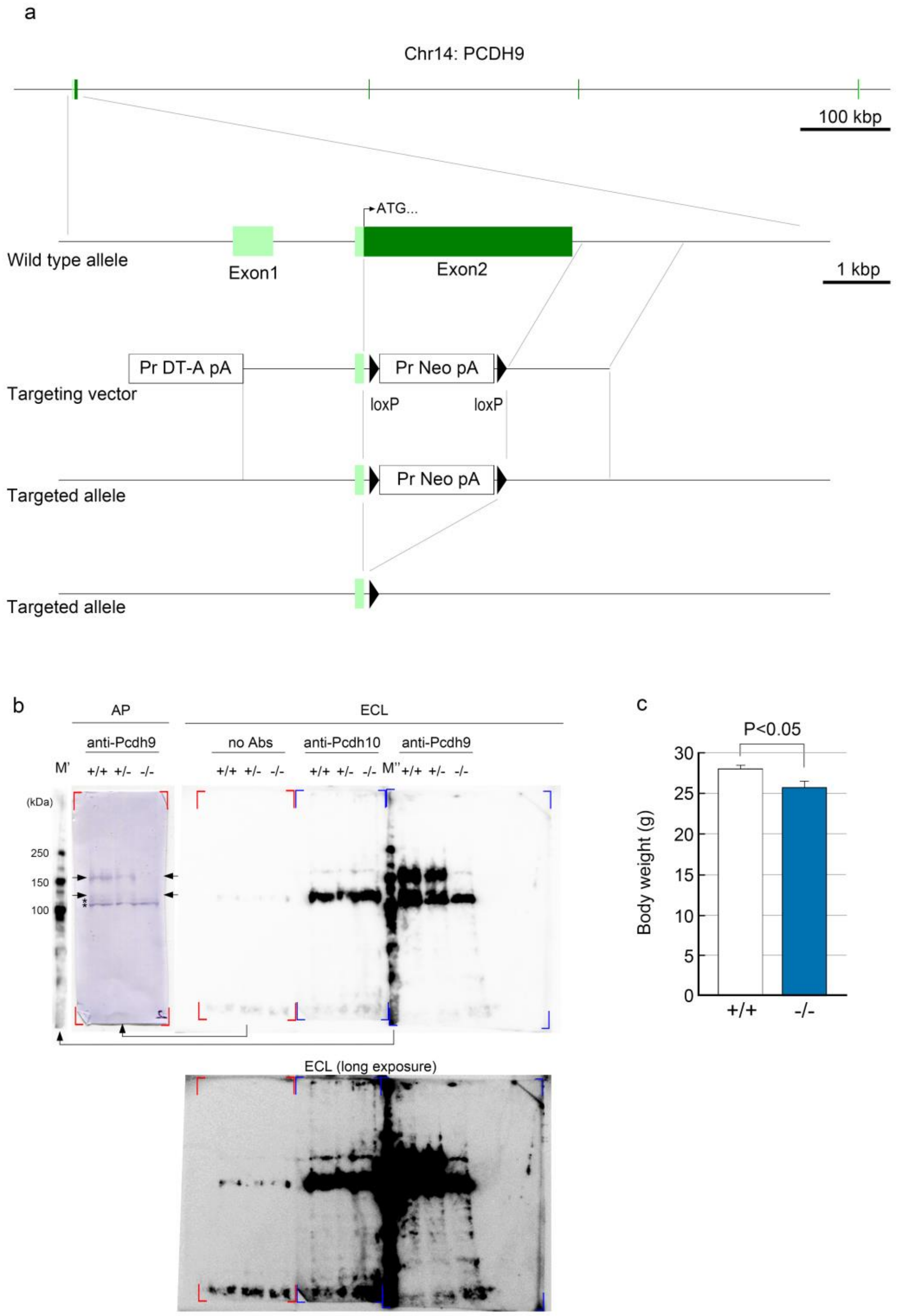

Supplementary Fig. S3 Production of the *Pcdh9* KO mice

(a) Target strategy for *Pcdh9* KO. The second exon of the *Pcdh9* gene including the start codon was replaced by the neomycin-resistant (neo) gene with a targeting vector. The targeted allele was then crossed with CAG-Cre mice, and the neomycin-resistant gene

was removed. (b) Western blot analysis showing the absence of Pcdh9 proteins but not Pcdh10 proteins in the Pcdh9 KO mice. For confirmation of the result of ECL, the negative-control filer with no antibody (marked red brackets) was incubated with anti-Pcdh9 and visualised with alkaline phosphatase (AP)-conjugated anti-rat Ig and Western blue (Promega). Arrows indicate the position of the Pcdh9 proteins whereas asterisks show nonspecific bands. The molecular markers marked M' and M" were derived from the same gel/filter except for different exposure times. +/+: wt, +/-: heterozygote, -/-: KO. (c) Bodyweight measurement of the *Pcdh9* KO mice.

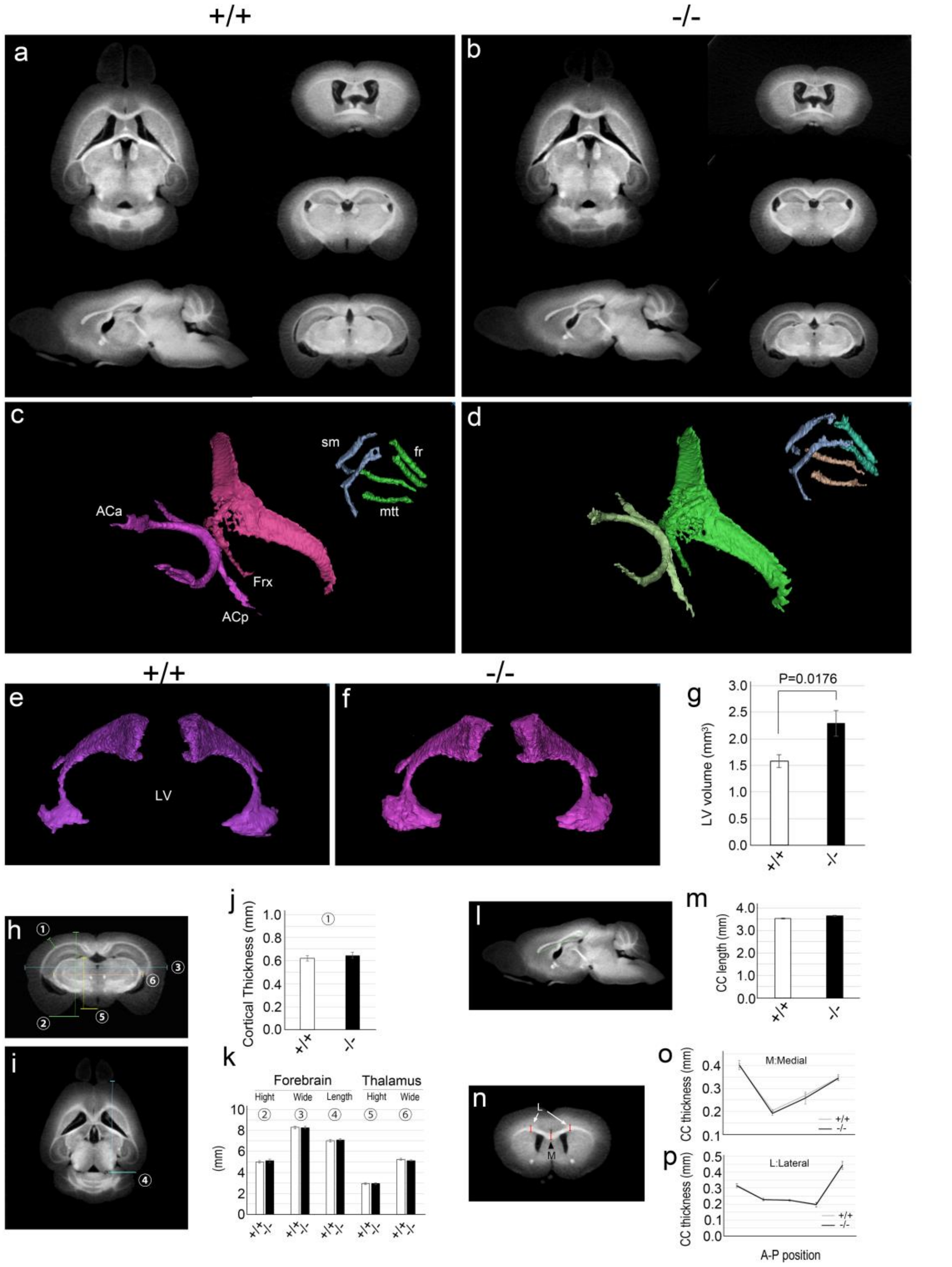

Supplementary Fig. S4 Overall brain structure of the *Pcdh9* KO mice

(a,b) Images of the brains with a CT scanner. (c,d) Three-dimensional reconstructions of some major fibre tracts (anterior commissure: ac, fornix: frx, stria medullaris: sm, fasciculus retroflexus:fr, and mammillothalamic tract: mtt). (e-g) Three-dimensional reconstructions of the ventricles. The volume of the ventricles of the *Pcdh9* KO mice is

larger than that of the wild-type (g). (h-p) Comparison of brain dimensions of the *Pcdh9* KO and wild-type mice in various regions including cortical thickness (j), size of forebrain and thalamus (k), and size of corpus callosum (cc; l-p).

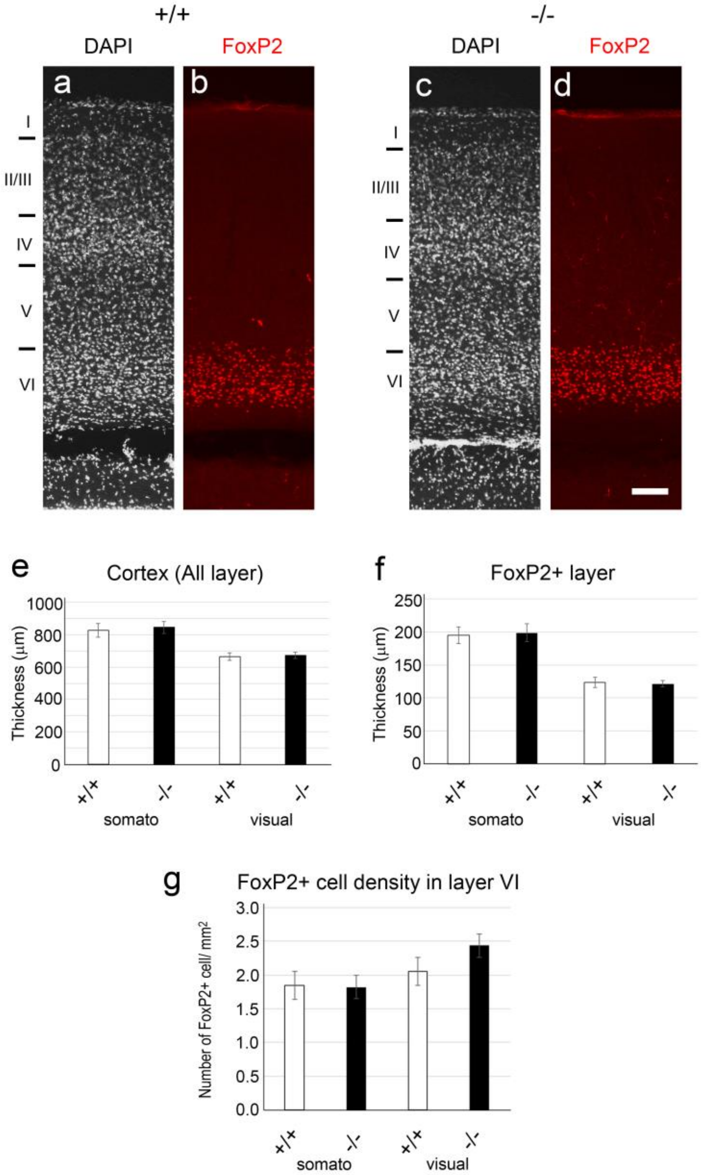

Supplementary Fig. S5 Cortical structure of the *Pcdh9* KO mice.
(a-d) Comparison of the lamina organisation of the cortex in the KO mice and the wild-type mice with staining of DAPI and anti-FoxP2. (e-g) Statistics of the cortical thickness of all layers (e) and FoxP2-positive deep layers (f), and density of $FoxP2^+$ cells (g). Scale bars, 100µm in (a-d).

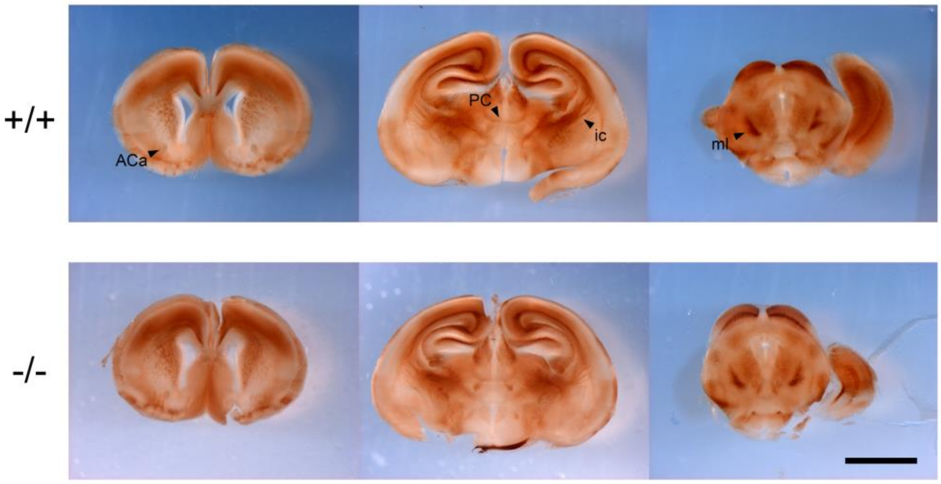

Supplementary Fig. S6 Major neural pathways in the *Pcdh9* KO mice.
Wild-type and *Pcdh9* KO mice brain slices were stained with an anti-neurofilament antibody. ACa: anterior part of anteriorcommissure, ic: internal capsule, ml: medial lemniscus, pc: posterior commissure.

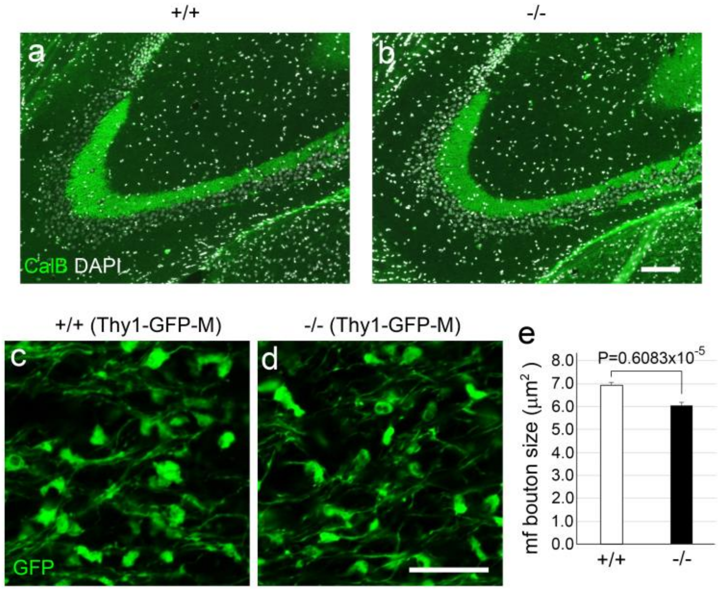

Supplementary Fig. S7 Terminals of the mossy fibres in the CA3 region of the *Pcdh9* KO mice.

(a,b) Immunofluorescent images of the CA3 region stained with anti-calbindin and DAPI. Mossy fibers in the CA3 regions are largely normal. (c,d) Synaptic boutons of the mossy fibres visualised in the Thy1 GFP M / Pcdh9 KO mouse stained with anti-GFP (e) Statistics of the bouton size of the mossy fibres. The size of the bouton in the *Pcdh9* KO mice is smaller than that of the wild-type mice. The size of bouton was measured with 397 boutons from five wt mice and 206 boutons from five *Pcdh9* KO mice.

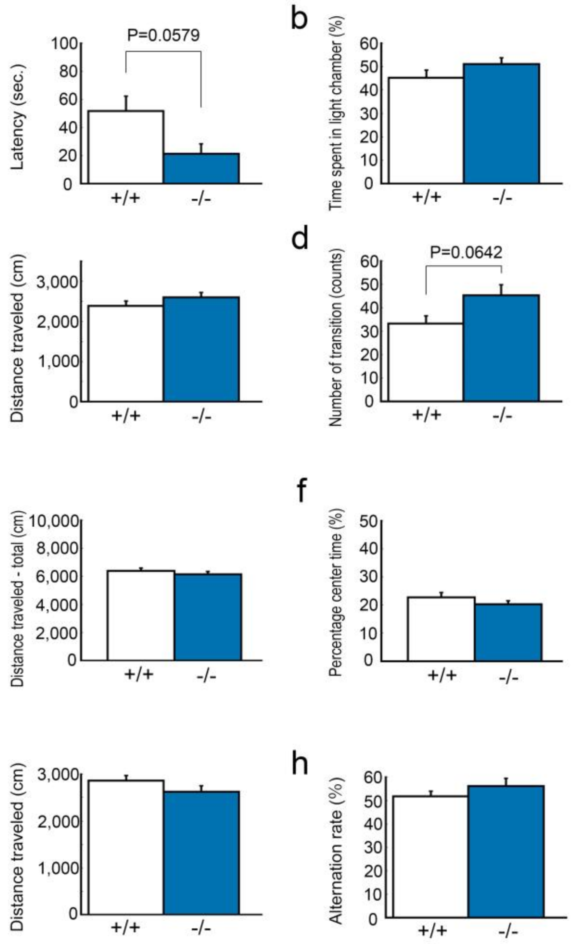

Supplementary Fig. S8 Behavior analyses of the *Pcdh9* KO mice in relation to fear emotion.

(a-d) In the light-dark transition test, the *Pcdh9* KO mice tended to enter the bright room with shorter latency (a) but they also tended to change the rooms more frequently (d). (e, f) In the open-field test, the *Pcdh9* KO mice behaved normally in every parameter except for distance traveled. (g, h) In the Y-maze test, no difference was observed in the change rate of arms and distance traveled between the *Pcdh9* KO mice and the wild-type mice.

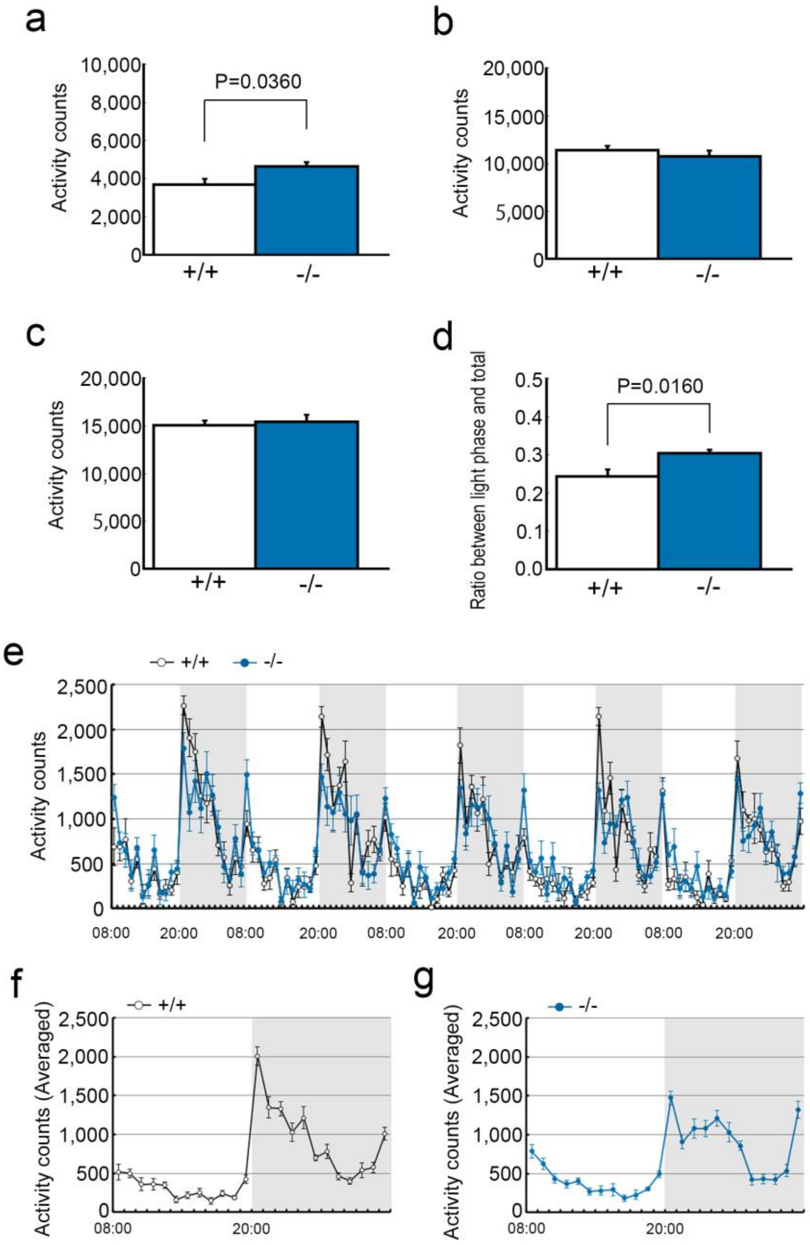

Supplementary Fig. S9 Home cage activity test of the *Pcdh9* KO mice.
(a-d) The activity of the *Pcdh9* KO mice in the light phases was higher than that of the wild type, and the ratio of activity in light/total activity was reduced in the *Pcdh9* KO mice. (e-g) Time course of the activities of the *Pcdh9* KO and the wild-type mice. Compared to the wild-type, the *Pcdh9* KO mice showed lower activity just after the transition from the light phase to the dark phase.

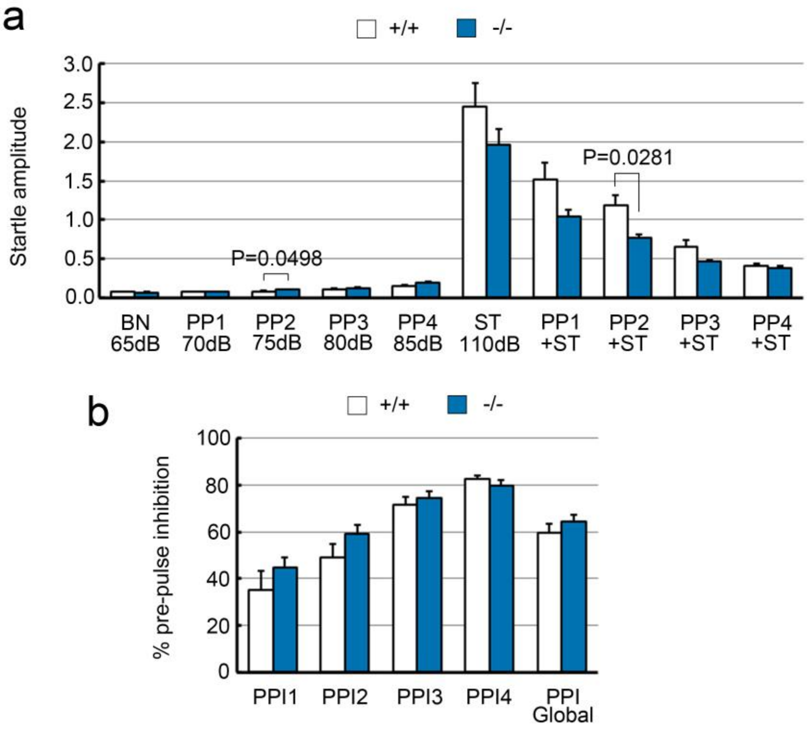

Supplementary Fig. S10 The pre-pulse inhibition test of the *Pcdh9* KO mice. (a) Startling amplitudes were generally normal except at PP2 75dB and PP2 +stimulation (ST) in the *Pcdh9* KO mice. (b) In the *Pcdh9* KO mice, pre-pulse inhibition was generally normal.

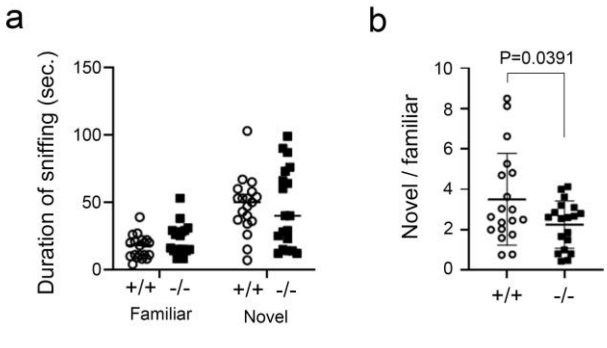

Supplementary Fig. S11 Object recognition test of the *Pcdh9* KO mice. (a) Duration of sniffing of the familiar vs novel objects. The difference between the genotypes was not evident. (b) The novel/familiar ratio of sniffing time. The ratio was lower in the *Pcdh9* KO mice than that of the wild-type mice.

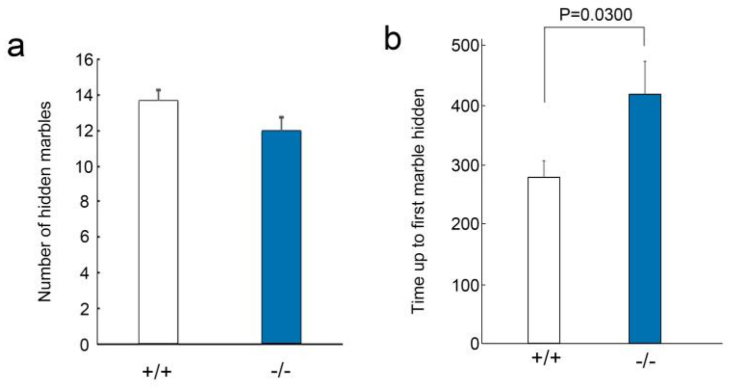

Supplementary Fig. S12 Marble burying test of the *Pcdh9* KO mice.
(a) The number of marbles hidden. (b) Latency to hide the first marble. The *Pcdh9* KO mice tended to delay the start of hiding as compared to the wild-type mice.
